# Ecological effects of *B. subtilis* C3 in kiwifruit rhizosphere soil and its prevention and control against root rot disease

**DOI:** 10.3389/fmicb.2025.1623463

**Published:** 2025-08-21

**Authors:** Xu Guoyi, Song Xiaolong, Ku Yongli, Tian Yuan, Li Ming, Cao Cuiling, Yu Huili, Si Peng

**Affiliations:** ^1^Zhengzhou Fruit Research Institute, Chinese Academy of Agricultural Sciences, Zhengzhou, Henan, China; ^2^Zhongyuan Research Center, Chinese Academy of Agricultural Sciences, Xinxiang, Henan, China; ^3^College of Horticulture of Henan Agricultural University, Henan, China; ^4^College of Landscape Architecture and Art of Henan Agricultural University, Zhengzhou, Henan, China; ^5^College of Forestry of Henan Agricultural University, Zhengzhou, Henan, China; ^6^College of Life Sciences, Northwest A&F University, Yangling, Shanxi, China

**Keywords:** Kiwifruit Root Rot, *Fusarium solani*, biocontrol, *Bacillus subtilis* C3, rhizosphere soil

## Abstract

As the world’s largest producer of kiwifruit, China faces significant yield and quality losses due to the widespread occurrence of kiwifruit root rot. To explore alternative biological control strategies for kiwifruit root rot, this study isolated 11 fungal isolates from diseased kiwifruit roots and identified *Fusarium solani* as the primary pathogen. Additionally, a biocontrol strain, *Bacillus subtilis* C3, was isolated from the rhizosphere of healthy kiwifruit and shown to significantly inhibit pathogen growth. The *B. subtilis* C3 strain effectively controls root rot via multiple mechanisms, including direct antagonism, secretion of antimicrobial proteins, promotion of seedling growth, and induction of plant defense enzymes. In pot and field trials, C3 treatment increased root fresh weight by 84.1%, enhanced root SOD and APX activities by 45.7 and 38.2%, respectively, and reduced disease severity. Moreover, C3 improved rhizosphere soil microbial diversity of the Rhizosphere, with the Shannon index increasing from 3.0 to 3.4. Unlike previous studies focusing solely on pathogen suppression, this work highlights the dual role of *B. subtilis* C3 in controlling root rot and restoring rhizosphere ecological function, offering a green and sustainable biocontrol strategy for kiwifruit production.

## 1 Introduction

Kiwifruit (*Actinidia chinensis*) is not only a delicious fruit but also rich in essential nutrients, providing various health benefits, including the treatment of cardiovascular diseases and other conditions (Satpal et al., 2021). In China, Shaanxi Province has become one of the key regions for kiwifruit cultivation, with the industry playing a significant role in the local economy and serving as a vital source of income for farmers (Wang et al., 2021). *Fusarium* species, particularly *F. solani*, are notorious soilborne pathogens that infect a wide range of crops and are frequently associated with root rot disease in kiwifruit and other economically important plants. These fungi invade plant root systems, leading to browning and rotting of root tissues, vascular discoloration, reduced nutrient uptake, and ultimately plant wilting or death (Kamali-Sarvestani et al., 2022). The pathogenicity of *Fusarium* is often linked to its ability to secrete cell wall-degrading enzymes and toxins, as well as to disrupt the balance of rhizosphere microbial communities and soil metabolite profiles, which further aggravates disease progression (Erazo et al., 2021). *Fusarium* pathogens can persist in soil as chlamydospores or infect plants through wound sites and natural openings, facilitating long-term survival and widespread transmission through contaminated soil or water (Bhar et al., 2021). Current control strategies rely on integrated approaches, including soil management, the use of resistant cultivars, crop rotation, and biological control agents such as *B. subtilis* to suppress *Fusarium* proliferation and mitigate disease impact (Ben Khedher et al., 2021; Attia et al., 2025). Most recently, in 2023, Wenpeng Song et al. identified *F. solani* and *F. breve* as the main pathogens responsible for kiwifruit root rot, using DNA sequence analysis (Song et al., 2023). The pathogens responsible for kiwifruit root rot are diverse, including species of Phytophthora, Pythium, and *Fusarium* (Feng et al., 2024). Current control measures for kiwifruit root rot include agricultural, physical, biological, and chemical methods. While these approaches have had some success in reducing root rot incidence, there is an increasing demand for more sustainable practices. As green agriculture gains traction and consumer demand for pesticide-free food rises, the use of biological control agents has gained significant attention (Abdelaziz et al., 2023). With rising living standards and greater awareness of sustainability, demand for nutrient-rich, non-polluting, and pesticide-free foods has also increased, leading to growing interest in biocontrol agents among farmers (Zhang F. et al., 2022). Consequently, numerous biological fungicides have been developed and are widely used to manage plant diseases. Biocontrol agents, such as bacteria, fungi, and actinomycetes, are now commonly applied in agricultural systems (Peng et al., 2021). Among these, *B. subtilis* stands out as a promising biocontrol agent due to its ability to produce a wide range of antimicrobial compounds and its remarkable resilience to harsh environmental conditions, such as high temperatures, drought, and UV radiation (Qiao et al., 2023). *B. subtilis* exerts its biocontrol effects through competitive exclusion of pathogens, the secretion of antibiotics, and the production of plant growth-promoting substances, such as auxins (Hu et al., 2025). These mechanisms help suppress plant diseases, promote plant growth, and reduce the reliance on chemical pesticides, making *B. subtilis* an ideal candidate for sustainable agricultural applications (Bonaterra et al., 2022).

This study aims to evaluate the potential of *B. subtilis* C3 as a biocontrol agent against kiwifruit root rot. We hypothesize that *B. subtilis* C3 can effectively inhibit the growth of *F. solani*, the primary pathogen responsible for kiwifruit root rot, and promote plant health. To test this hypothesis, we will conduct *in vitro* antagonism tests, greenhouse trials, and field applications to assess the efficacy of *B. subtilis* C3 in controlling root rot. The goal is to provide an environmentally friendly alternative to chemical pesticides, supporting the development of sustainable agricultural practices.

## 2 Materials and methods

### 2.1 Isolation and identification of kiwi root rot pathogens

Disease samples were collected from a kiwifruit root rot orchard in Meixian, Shaanxi Province (Supplementary Figure S1). The samples were washed with tap water to remove soil, sterilized with 75% ethanol for 30 s, and rinsed thoroughly with sterile distilled water. Using the tissue separation method, diseased roots were cut into small pieces, excess moisture was absorbed with filter paper, and the tissue pieces were placed on sterilized Potato Dextrose Agar (PDA) (Shanghai Maokang Biotechnology Co., Ltd., Shanghai, China) solid medium and incubated for 3 days. All procedures were performed under sterile conditions. Mycelium was transferred to fresh medium and incubated for 2 days, then subcultured until no contaminants were observed. Finally, purified cultures were transferred to sterilized PDA slants (prepared by pouring sterilized PDA into test tubes and solidifying at an angle before cooling to create a slanted surface for increased mycelial growth area) and stored at 4°C (Wang et al., 2020). The pathogenicity of the isolated fungi was evaluated on 2-year-old “Xuxiang” kiwifruit using shoots and leaves. Fungi were grown on PDA for 7 days, and spores were collected and diluted in 0.05% Tween sterile water. The root tissue of kiwi seedlings was soaked in spore suspension for 12 h, then rinsed, with daily observations of the changes. Roots were similarly treated after cleaning. A control was established with Tween solution alone. The experiment was conducted at 25°C with a 12-h light/dark cycle, repeated five times. The fungal pathogen was preliminarily identified based on both colony morphology and microscopic characteristics. Infected root tissues were surface-sterilized, sectioned, and placed on potato dextrose agar (PDA) medium for fungal isolation. The resulting colonies were observed for morphological features. A microscopic examination was performed to observe hyphal and conidial structures under a light microscope. For molecular identification, genomic DNA was extracted from pure fungal cultures. The internal transcribed spacer (ITS) region was amplified by PCR using the universal fungal primers ITS1 (TCCGTAGGTGAACCTGCGG) and ITS4 (TCCTCCGCTTATTGATATGC). PCR was conducted using Superstar PCR Mix (Kangrun, China). The amplified products were purified using the AXYGEN DNA Gel Purification Kit and sequenced using a 3730xl DNA Analyzer (ABI, USA). The obtained sequence was compared with sequences in the GenBank database using BLAST analysis.

### 2.2 Biological characteristics of *B. subtilis* C3 strain

Fifteen rhizosphere soil samples were collected from healthy kiwifruit orchards. Each sample was subjected to heat treatment at 90°C for 20 min. Then, 2 g of soil was suspended in sterile distilled water, serially diluted, and spread onto Luria-Bertani (LB) agar plates. After incubation, colonies exhibiting typical Bacillus-like morphology and Gram-positive staining were selected. Five *isolates* were obtained for further analysis. These isolates were screened for antagonistic activity against kiwifruit root rot pathogens using a dual-culture assay. The isolate showing the strongest inhibition was chosen for molecular identification. The 16S rRNA gene (~1,500 bp) was amplified using universal primers: forward primer AGAGTTTGATCCTGGCTCAG and reverse primer CTACGGCTACCTTGTTACGA. The PCR product was sequenced and subjected to BLAST analysis. The phylogenetic tree of the selected isolate was constructed using the Maximum Likelihood method with the Tamura-Nei substitution model and the Nearest-Neighbor-Interchange (NNI) heuristic algorithm in MEGA11 software.

The enzymatic activity of *B. subtilis* C3 was measured as shown in Supplementary Figure S4. Amylase, chitinase, and cellulase production were quantified using the 3,5-dinitrosalicylic acid (DNS) method (Pujiati Ardhi et al., 2021); the quantitative determination of protease activity was conducted using the aniline blue colorimetric method (Medison et al., 2023); phytase production was measured by the molybdate-vanadate colorimetric assay (Carmo-Silva et al., 2024); and phosphatase activity was assessed using the p-nitrophenyl phosphate (pNPP) method (Gao et al., 2022). The secretion of auxin by *B. subtilis* C3 was determined by the Salkowski colorimetric assay (Azarmi et al., 2015), and gibberellin secretion was assessed using fluorescence assays (Li et al., 2021).

### 2.3 Study on the mechanism of C3 controlling kiwifruit root rot

Antagonistic activity of *B. subtilis* C3 against kiwifruit root rot pathogens was assessed using the mycelial plug confrontation method. Activated C3 colonies were cultured, and agar plugs were placed 1.5 cm from the pathogen inoculation site on modified PDA plates. After 2 days of incubation at 30°C in darkness, inhibition zones were photographed and measured (Li Y. et al., 2023). To evaluate antimicrobial protein activity, crude extracts were obtained by ammonium sulfate precipitation at varying saturations. The Oxford cup method was used to test inhibition against the pathogen. Pathogen spores were inoculated (2%) into PDA medium and poured into plates. Sterilized Oxford cups were filled with protein extracts; phosphate buffer served as a control. Plates were incubated at 25°C for 3 days, and inhibition zone diameters were recorded. Each treatment was conducted in triplicate (Duan et al., 2023).

To evaluate the effect of *B. subtilis* C3 on the promotion of kiwifruit seedling growth, germinated seeds were immersed in a 0.1% gibberellin solution and stored at 4°C for 36 h. A tissue culture flask containing 25 g of sterilized organic soil was inoculated with 1 × 10^8 CFU/g of *B. subtilis* C3 fermentation broth and thoroughly mixed. A control group without inoculation was also included. Each treatment was repeated 10 times. The seeds were then planted in the culture flask and incubated for 35 days under conditions of 20% water content, 27°C, and a 12-h light/dark cycle. Biomass, morphological parameters, and growth-related indicators were measured.

Chlorophyll content was determined using the 80% acetone extraction method. Briefly, 0.2 g of fresh leaf tissue was ground in 80% (v/v) cold acetone, and the homogenate was centrifuged at 12,000 rpm for 10 min at 4°C. The absorbance of the supernatant was measured at 663 nm and 645 nm using a UV–visible spectrophotometer. Chlorophyll a, chlorophyll b, and total chlorophyll contents were calculated using the Arnon equations (Carmo-Silva et al., 2024). Soluble sugar content was analyzed by the anthrone–sulfuric acid method. In brief, 0.2 g of leaf tissue was extracted in 5 mL of distilled water at 100°C for 30 min. The extract was centrifuged, and 1 mL of the supernatant was reacted with 5 mL of freshly prepared anthrone reagent (0.1% anthrone in concentrated sulfuric acid). The reaction mixture was incubated in a boiling water bath for 10 min, then cooled, and absorbance was recorded at 620 nm, Glucose was used as the standard for quantification (Gao et al., 2022). Soluble protein content in leaf tissue was measured using the Bradford assay. Approximately 0.2 g of fresh leaf sample was homogenized in 5 mL of phosphate buffer (pH 7.0), and the extract was centrifuged at 12,000 rpm for 10 min. An aliquot of the supernatant was mixed with Coomassie Brilliant Blue G-250 reagent, and the absorbance was read at 595 nm. Bovine serum albumin (BSA) was used as the standard (Deans et al., 2018).

The activities of resistance enzymes, including polyphenol oxidase (PPO), phenylalanine ammonia lyase (PAL), and superoxide dismutase (SOD), were measured in 30-day-old kiwifruit seedlings. Ascorbate peroxidase (APX) activity was determined by monitoring the change in absorbance at 290 nm, with one unit of enzyme activity (U) defined as a 0.01 change in absorbance per minute (Azarmi et al., 2015).

### 2.4 Field experiments of applying C3 into kiwifruit rhizosphere soil

The freshly collected rhizosphere soil from root rot-infected kiwifruit trees was directly used in a small pot experiment without air drying. The experimental setup included three treatments: (1) unplanted soil (H), (2) rhizosphere soil from root rot-infected kiwifruit trees (CK), and (3) rhizosphere soil from root rot-infected kiwifruit trees mixed with *B. subtilis* C3 at 1.8 g/kg soil (T). Each treatment was replicated six times, and kiwifruit seedlings with two true leaves were transplanted into the pots. After 30 days of growth, seedling growth was observed, and biomass and morphological indices were measured.

The experiment was conducted in a 15-year-old kiwifruit orchard located in Meixian, China (latitude E107.81°, longitude N34.21°), a region characterized by a temperate continental monsoon climate. Meixian is a standardized kiwifruit production demonstration area, with an average annual temperature of 12.9°C and an average annual rainfall of 609.5 mm. To evaluate the effect of bio-control agents on soil microbial biomass, nutrients in continuous cropping soils, and kiwifruit fruit quality, 10 diseased trees with root rot were randomly selected. Healthy trees served as the absolute control, and the diseased trees were the control. C3 microbial agent (500 g/tree) was applied around the tree trunk (0.5 m radius, 0.2 m soil depth). Soil samples were collected from four different sites within the application range (0–20 cm depth) at five stages. Fresh soil samples were used for microbial counts (plate dilution method) and microbial community analysis (Biolog ECO-plate method) (Li et al., 2021). The remaining soil was air-dried, sieved (2 mm), and analyzed for soil nutrients.

At the fruit maturation stage, leaves and fruits were collected from uniform heights and four orientations (east, south, west, and north) of each plant to assess stress-related enzymatic activities and fruit quality, ensuring similar fruit shape and weight. For polyphenol oxidase (PPO), 0.5 g of fresh leaf tissue was homogenized in 5 mL of 0.1 mol/L phosphate buffer (pH 6.8). After centrifugation at 12,000 × g for 15 min at 4°C, the supernatant was reacted with 0.1 mol/L catechol, and absorbance was recorded at 420 nm. PPO activity was expressed as the change in absorbance per minute at 420 nm. Phenylalanine ammonia-lyase (PAL) activity was measured by incubating the enzyme extract with 20 mmol/L L-phenylalanine in 0.05 mol/L borate buffer (pH 8.8), and absorbance was recorded at 290 nm. One unit of PAL activity (U) was defined as an increase of 0.01 in absorbance at 290 nm per hour. Catalase (CAT) activity was determined by measuring the decomposition rate of 50 mmol/L H₂O₂, with absorbance monitored at 240 nm. One unit of CAT activity (U) was defined as a decrease of 0.01 in absorbance per minute. Ascorbate peroxidase (APX) activity was assessed by monitoring the oxidation of 0.5 mmol/L ascorbic acid in the presence of 0.1 mmol/L H₂O₂. The decrease in absorbance at 290 nm was recorded, and one unit of APX activity (U) was defined as a decrease of 0.01 in absorbance per minute.

The serial dilution method was applied to assess the biomass of microorganisms. The microorganism suspension was inoculated onto LB solid medium to test bacterial biomass, onto Rose Bengal medium to test fungal biomass, and onto Actinomycetes Isolation Agar to quantify the number of actinomycetes (Castle et al., 2011). Biolog ECO-plate method was applied to test the change of microbiology biomass (Classen et al., 2003). The activity of 5 enzymes in soil under different treatments was determined: sucrase, phosphatase, protease, urease and polyphenol oxidase. The activity of sucrase was measured by DNS method (Xiao et al., 2020). Phosphatases was measured by p-nitrophenyl phosphate method (Tabatabai and Bremner, 1969). Protease was measured as using the method offered by Ladd and Butler (Ladd and Butler, 1972). Urease was assayed by indophenol blue colorimetric method (Yang et al., 2022). Polyphenol oxidase was assayed by epigallocatechin colorimetric method (Tabatabai and Fu, 2021). Soil pH, total nitrogen, nitrate nitrogen, ammonium nitrogen, available phosphorus, available potassium, and organic matter content were determined.

### 2.5 Statistical analysis

Data analysis and visualization were performed using Microsoft Excel 2013, PS, Origin 8.0, and GraphPad Prism 10. Statistical analyses, including significance testing of differences, were conducted using SPSS 23.0 with the least significant difference (LSD) method.

## 3 Results

### 3.1 Biochemical properties and biocontrol mechanisms of *B. subtilis* C3

According to the isolation and identification results, colonies grown on PDA were initially white and gradually turned pinkish with time, displaying a cottony texture and irregular margins. Microscopic observations revealed septate hyphae and fusiform macroconidia with morphological characteristics typical of *Fusarium* species as shown in Supplementary Figure S2. Under light microscopy (scale bar = 50 μm), numerous crescent-shaped or slightly curved fusiform conidia were observed, varying in size, with some being multi-septate. The conidia were formed at the tips of conidiophores, appeared hyaline or lightly blue-stained, and exhibited clear structure with typical morphological characteristics of the genus *Fusarium*, as shown in Supplementary Figure S3. PCR amplification of the ITS region produced a clear band of approximately 500 bp. Sequencing analysis showed that the amplified fragment was 568 bp in length and shared 99% sequence similarity with *Fusarium solani* (GenBank accession no. OR123393), confirming the pathogen as *F. solani*. Based on these morphological and molecular identification results, *F. solani* was confirmed as the causal agent of root rot in “Xuxiang” kiwifruit, Supplementary Figure S4 Phylogenetic tree of *F. solani* based on ITS region sequences.

Among the five bacterial isolates, one strain showed the most significant antagonistic activity against the kiwifruit root rot pathogen, as shown in Supplementary Figure S5. Sequencing results revealed that the amplified 16S rRNA gene fragment had 99% identity with *B. subtilis* strain SBRh5 (GenBank accession no. HQ443229.1). The strain was designated as *B. subtilis* C3 and preserved in the laboratory, with the GenBank accession number KY983582. The phylogenetic tree confirmed the taxonomic position of the isolate as *B. subtilis*, as shown in Supplementary Figure S6.

**Figure 1:**
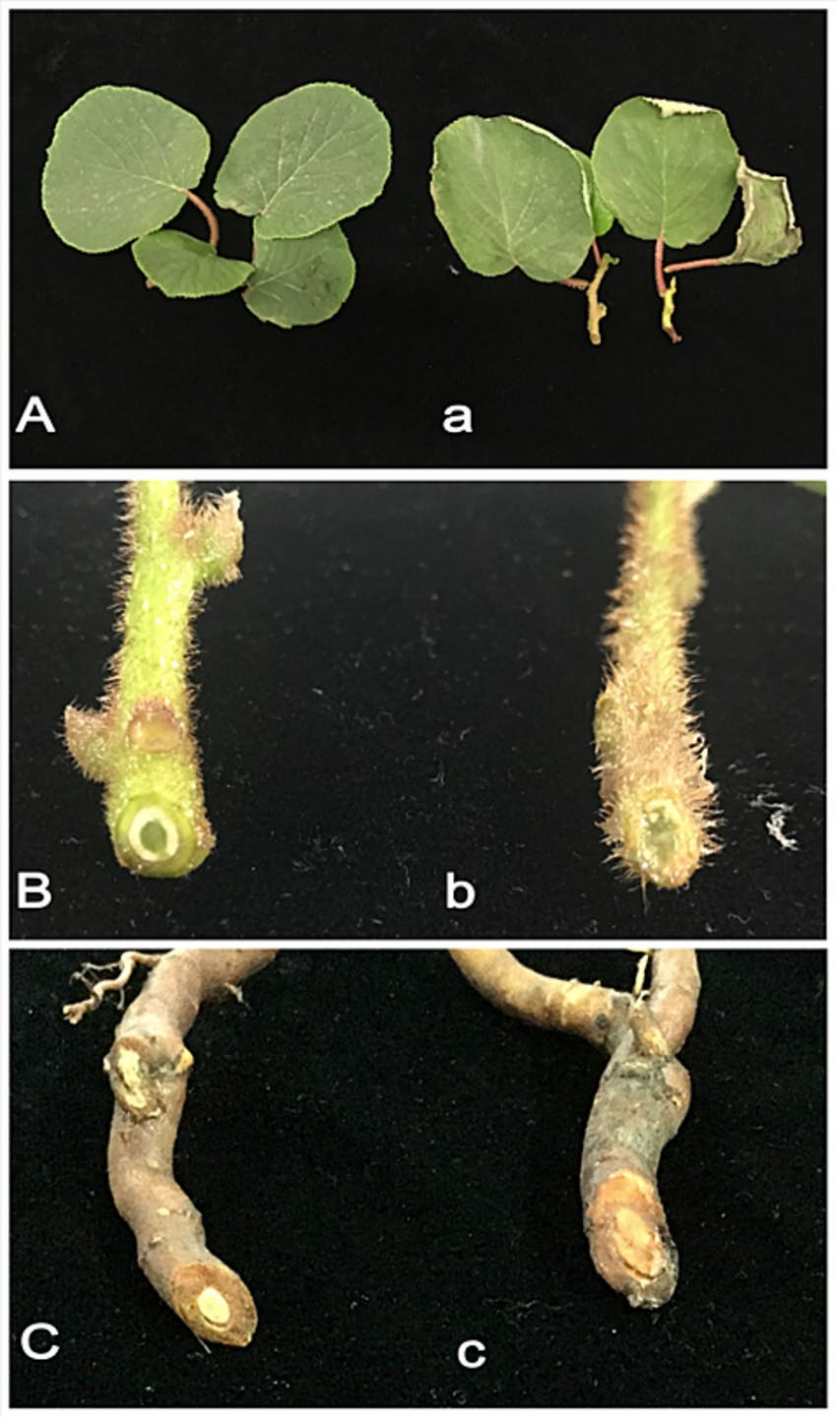
Symptoms of kiwifruit inoculated with pathogenic *Fusarium solani*. **(Aa)** After 3 days of inoculation, wilting symptoms appeared on the leaves. **(Bb)** After 5 days of inoculation, the shoots exhibited visible signs of rot. **(Cc)** After 7 days of inoculation, root rot symptoms became evident. **(A–C)** Control group. **(a–c)** Inoculated with the pathogen.

Root segments from kiwifruit plants exhibiting root rot symptoms were surface sterilized and cultured on PDA medium, resulting in the isolation of 11 fungal isolates. Each isolate was tested for pathogenicity by inoculating its spore suspension onto healthy kiwifruit branches, leaves, and roots. Only one isolate induced consistent disease symptoms: after 3 days, leaves showed dehydration and wilting (Figure 1Aa); after 6 days, branches exhibited wilting and vascular tissue damage (Figure 1Bb); after 10 days, root xylem browned, showing a clear difference from the control (Figure 1Cc).

Supplementary Table S1 highlights the enzyme-producing capabilities of *B. subtilis* C3. The strain exhibited significant amylase activity (2.08 U/mL) and cellulase activity (180.14 U/mL) after 24 h in LB medium. It also demonstrated protease and chitinase production, along with phosphatase (12.49 U/mL) and phytase (1.81 U/mL) activities, indicating its potential for organic phosphorus solubilization. Qualitatively confirmed enzyme production, including amylase, cellulase, protease, phosphatase, and chitinase, as shown in Supplementary Figure S7.

As shown in Supplementary Figure S8, *B. subtilis* C3 demonstrated notable antagonism against the pathogen, forming a 0.79 cm inhibition zone, likely through nutrient competition, ecological niche exclusion, and suppression of spore germination and mycelial growth. Proteins precipitated with 50% saturated ammonium sulfate exhibited stronger inhibition (2.05 cm inhibition zone), while other protein fractions showed negligible effects. The production of chitinase by *B. subtilis* C3 suggested it may play a pivotal role in its inhibitory activity.

**Figure 2:**
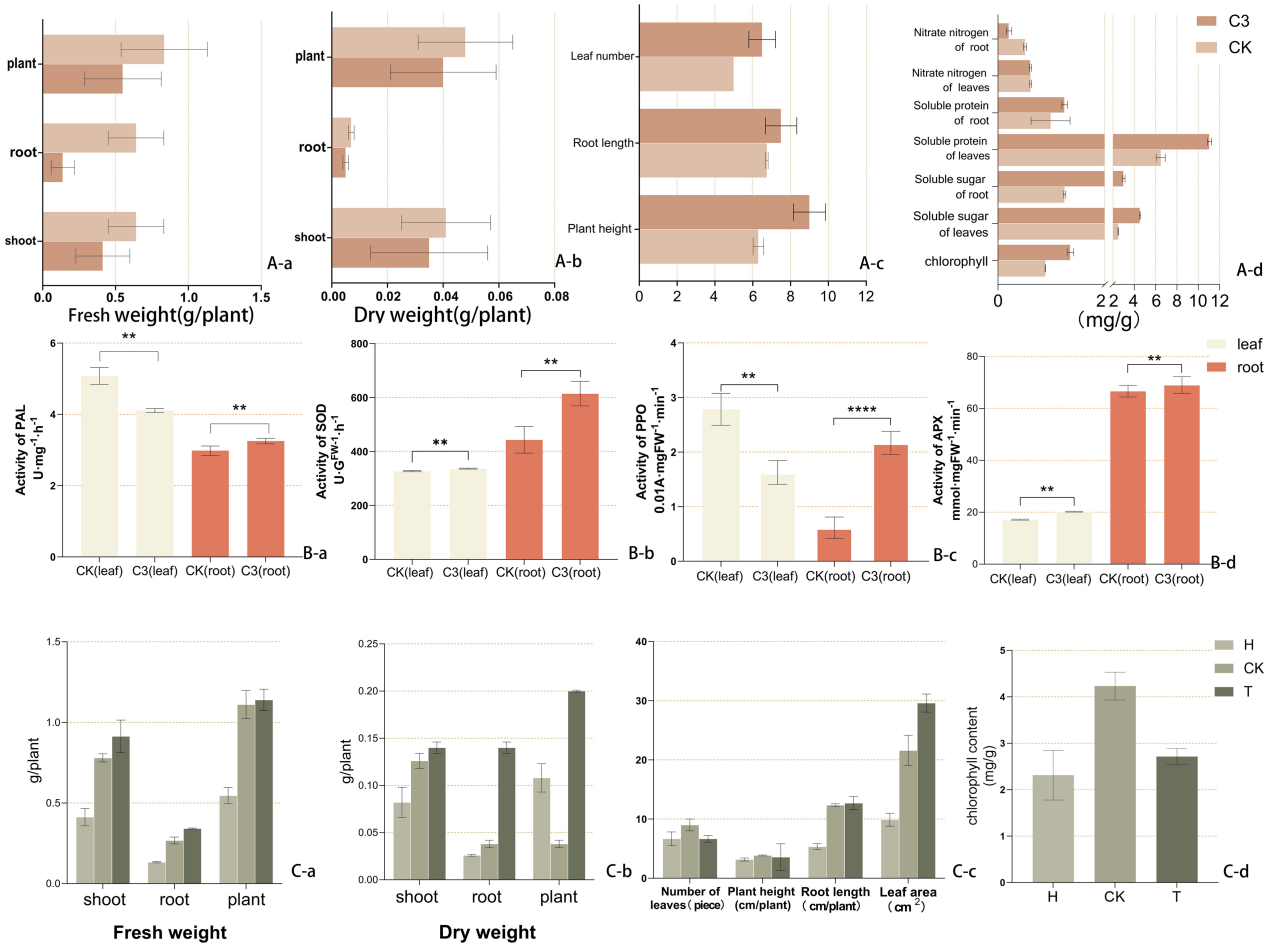
Effects of *Bacillus subtilis* C3 treatment on the biomass of kiwifruit seedlings. **(Aa)** Fresh weight. **(Ab)** Dry weight. **(Ac)** Morphological indicators. **(Ad)** Morphological indicators. **(Ad)** Physiological indicators. **(Ba–d)** Phenylalanine ammonia lyase (PAL), superoxide dismutase (SOD), polyphenol oxidase (PPO), and ascorbate peroxidase (APX). **(Ca)** Fresh weight. **(Cb)** Dry weight. **(Cc)** Growth traits. **(Cd)** Chlorophyll content. Soil from the previous wheat crop (H), Soil from the rhizosphere of kiwifruit plants with root rot disease (CK), Soil from the rhizosphere of kiwifruit plants with root rot disease treated with C3 biocontrol agent (1.8 g/kg soil). Asterisks above the columns indicate statistically significant differences between treatments, as determined by one-way ANOVA followed by Tukey’s *post-hoc* test. Specifically, *p* < 0.05 is denoted by *, *p* < 0.01 by **, and *p* < 0.001 by ***. Columns without asterisks indicate no significant difference (*p* ≥ 0.05) compared to the control group.

Supplementary Table S2 showed the hormone secretion ability of strain C3, which produced 24.263 mg/L of auxin and 0.041 mg/L of gibberellin. These hormones were secreted into the soil, promoting the growth of kiwifruit seedlings. Figures 2Aa,b illustrated the effect of C3 treatment on kiwifruit seedling biomass. These results indicated that *B. subtilis* C3 significantly enhanced biomass accumulation in kiwifruit seedlings, with shoot, root, and total fresh weight increasing by 55.45, 39.86, and 1.64%, respectively, compared to the CK group, thereby improving overall plant growth and productivity. Figure 2Ac demonstrated that after C3 treatment, plant height, root length, and leaf area increased by 42.86, 11.11, and 23.08%, respectively, compared to the untreated group. Figure 2Ad further revealed that the addition of *B. subtilis* C3 significantly improved related growth parameters, including chlorophyll content, soluble sugar, and soluble protein, in kiwifruit seedlings.

### 3.2 Effect of C3 treatment on soil microbial activity and diversity

Supplementary Figure S9 showed the effects of different treatments on the morphology of kiwifruit seedlings based on the results of the pot experiment. In Figure 2Ca, the shoot fresh weight and total plant fresh weight of seedlings in the T group were 0.914 and 1.140 g, respectively, which were 121.40 and 108.80% higher than those in the H group, with no significant difference compared to the CK group. The root fresh weight in the T group was significantly higher than that in the CK and H groups, being 1.28 times and 2.57 times greater, respectively. In Figure 2Cb, the shoot dry weight and total plant dry weight in the T group were 0.140 and 0.200 g, respectively, which were 70.73 and 85.19% higher than those in the H group, with no significant difference compared to the CK group. The root dry weight in the T group was significantly higher than that in the CK and H groups, being 1.39 times and 2.04 times greater, respectively. In Figure 2Cc, the root length of seedlings in the T group was 138.14% higher than that in the H group, with no significant difference compared to the CK group. However, the leaf area of seedlings in the T group was significantly higher than that in the H and CK groups, being 2.99 times and 1.37 times greater, respectively. Figure 2Cd shows the chlorophyll content in the leaves of kiwifruit seedlings under different treatments. The T group exhibited a significantly higher chlorophyll content than the H group, with an increase of 15.90%, although it remained significantly lower than that of the CK group.

**Figure 3:**
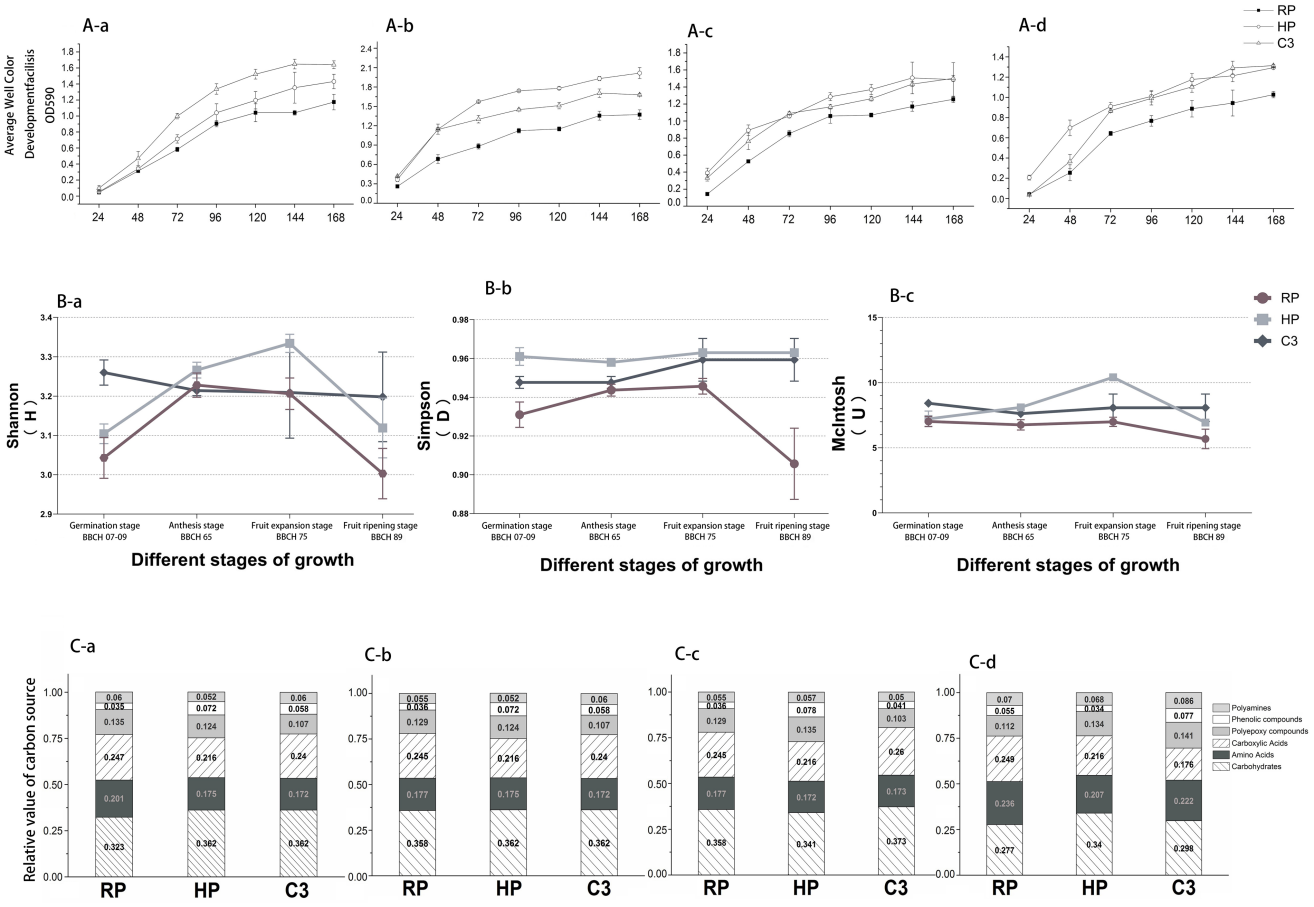
different treatments on soil microbial activity, diversity, and carbon source utilization across kiwifruit growth stages. **(Aa)** Germination stage (BBCH 07–09). **(Ab)** Anthesis stage (BBCH 65). **(Ac)** Fruit expansion stage (BBCH 75). **(Ad)** Fruit ripening stage (BBCH 89). HP, Healthy fruit trees; RP, Kiwifruit with root rot disease; C3, C3-treated kiwifruit with root rot. **(Ba)** Shannon index. **(Bb)** Simpson index. **(Bc)** McIntosh index. **(Ca)** Germination stage (BBCH 07–09). **(Cb)** Anthesis stage (BBCH 65). **(Cc)** Fruit expansion stage (BBCH 75). **(Cd)** Fruit ripening stage (BBCH 89).

First, it was found that the biomass of c3 treated kiwifruit root rot seedlings increased significantly through the results of the growth promotion pot experiment, and then field experiments were conducted. This was followed by field experiments. Soil microbial activity, measured as AWCD (Average well color development was used to evaluate the overall metabolic activity of microbial communities in the soil samples. It reflects the capacity of microorganisms to utilize a variety of sole carbon sources present in the Biolog ECO microplate.), varied significantly across treatments (C3, HP, RP) and kiwifruit growth stages (Figure 3A). During the Germination stage (BBCH 07–09), AWCD in the C3 treatment became significantly higher than HP and RP after 72 h and remained superior at 96 h, with RP showing the lowest activity (Figure 3Aa). At the Anthesis stage (BBCH 65), although AWCD of C3 was higher than HP at 72 h, its overall activity was lower than HP (Figure 3Ab). The Fruit expansion stage (BBCH 75) exhibited the highest microbial activity among all growth stages, with initial AWCD (24 h) higher than other periods (Figure 3Ac). After 48 h, AWCD of HP surpassed C3, showing the highest activity during this stage, while RP remained the lowest. During fruit ripening, C3 exhibited a significant increase in AWCD between 48 and 72 h, though it was still lower than HP (Figure 3Ad). Overall, C3 and HP treatments enhanced soil microbial activity compared to RP, with C3 significantly promoting inter-root microbial activity to benefit kiwifruit root rot management.

The Shannon index of RP was significantly lower than HP and C3 across all growth stages (*p* < 0.05), with C3 showing the highest values (*p* < 0.05), as shown in Figure 3Ba. At the flowering stage, no significant differences were observed among treatments. The Simpson’s index of HP peaked during the Fruit ripening stage (BBCH 89), while both C3 and RP declined at this stage (Figure 3Bb). The McIntosh index of RP decreased continuously from germination to the Fruit ripening stage (BBCH 89). HP and C3 showed the highest McIntosh index at the Fruit expansion stage (BBCH 75), followed by a decline at the Fruit ripening stage (BBCH 89), but remained significantly higher than RP (*p* < 0.05), as illustrated in Figure 3Bc. The microbial functional diversity index of root rot–affected soils treated with biocontrol fungal fertilizer exhibited a pattern like that observed in healthy soils.

The Biolog-ECO plate analysis revealed changes in the relative utilization of six groups of carbon sources during different kiwifruit growth stages. During the Germination stage (BBCH 07–09), the relative utilization of carboxylic acids and amino acids was higher in all treatments (Figure 3Ca), with RP showing the highest utilization of polyamines, carboxylic acids, and amino acids, while C3 exhibited higher sugar utilization compared to HP and RP. At the flowering stage (Figure 3Cb), sugar utilization increased across all treatments, while carboxylic acids and amino acids utilization decreased. RP showed the highest utilization of polymers, polyamines, carboxylic acids, and amino acids, but lower utilization of phenolics compared to HP and C3. During the Fruit expansion stage (BBCH 75), C3 exhibited the highest relative utilization of sugars and carboxylic acids (Figure 3Cc), while HP showed the highest utilization of polyphenolic compounds, polyamines, and polymers. In the Fruit ripening stage (BBCH 89), the utilization of sugars by RP and C3 decreased significantly (Figure 3Cd), while amino acid utilization increased. HP exhibited reduced utilization of phenolic compounds, while C3 showed enhanced utilization of polyphenolic compounds and polyamines.

**Figure 4:**
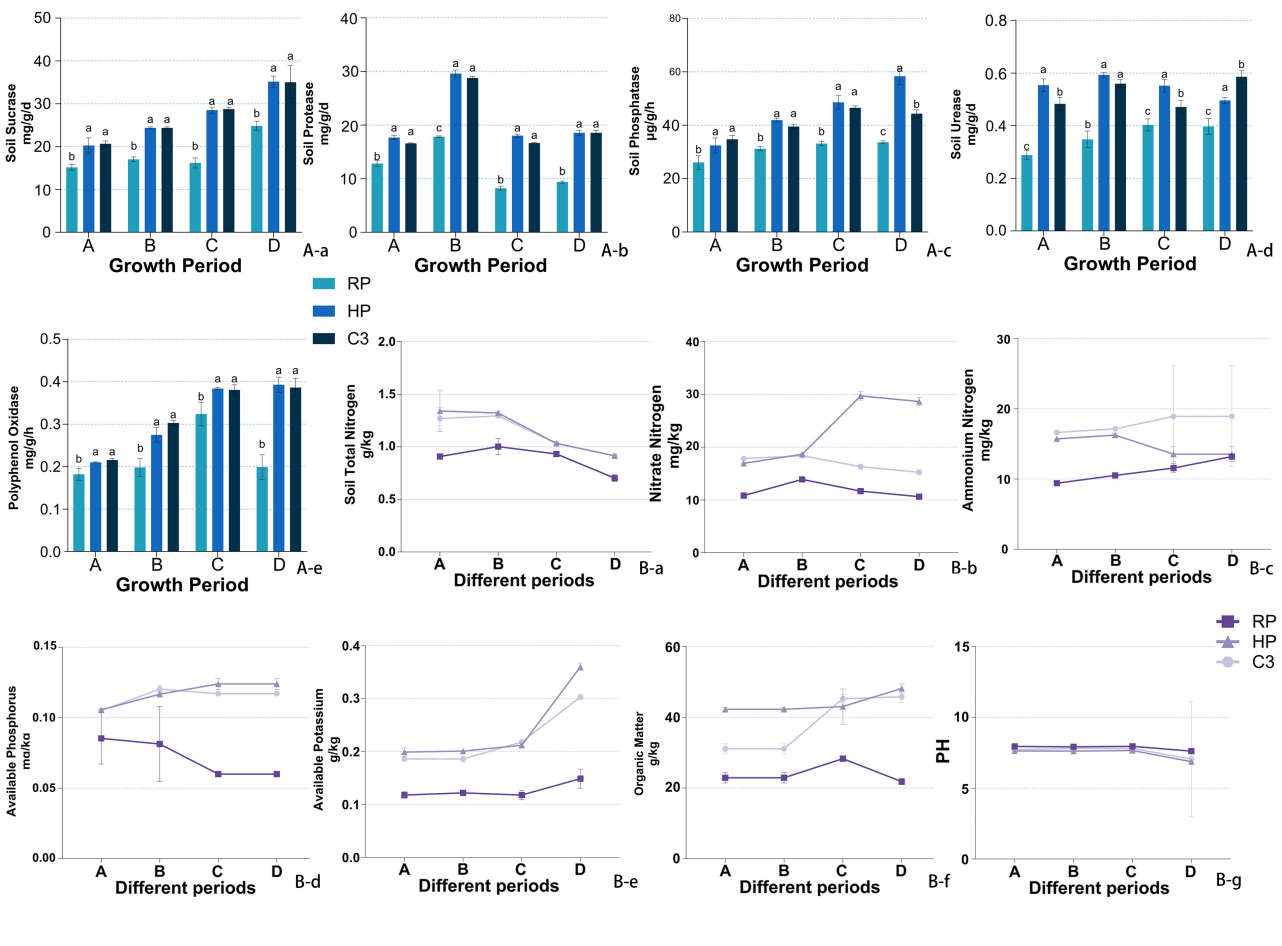
The effects of different fertilizer treatments on soil enzyme activities and their influence on soil physical and chemical properties. **(Aa)** Sucrase activity. **(Ab)** Protease activity. **(Ac)** Phosphatase activity. **(Ad)** Urease activity. **(Ae)**: Polyphenol oxidase activity. **(Ba)** Total nitrogen. **(Bb)** Nitrate nitrogen. **(Bc)** Ammonium nitrogen. **(Bd)** Available phosphorus. **(Be)** Available potassium. **(Bf)** Organic matter. **(Bg)** Soil PH. **(A)** Germination stage (BBCH 07–09), **(B)** anthesis stage (BBCH 65), **(C)** fruit expansion stage (BBCH 75), **(D)** fruit ripening stage (BBCH 89). In the figure, labels like “A-a” and “B-b” denote specific measured indicators, where capital letters **(A, B)** indicate indicator categories (soil enzymes and soil nutrients), and lowercase letters (a, b, c…) distinguish individual indicators within each category.

Throughout the kiwifruit growth cycle, the activities of sucrase, protease, polyphenol oxidase, phosphatase, and urease in the inter-root soils were significantly higher in the healthy fruit tree group (HP) compared to the root rot diseased tree group (RP) (Figure 4A). Sucrase activity in the C3 group was significantly higher than that of the RP group across all growth stages, with increases of 33.89, 43.00, 76.13, and 41.76% during the germination, flowering, fruit expansion, and Fruit ripening stage (BBCH 89), respectively (Figure 4Aa). There was no significant difference between the C3 and HP groups. Protease activity in the C3 group was 37.81–118.87% higher than that of RP throughout the growth cycle and was comparable to that of the HP group, with no significant differences observed between C3 and HP (Figure 4Ab). Phosphatase activity in the C3 group was significantly higher than that in RP at all stages and was comparable to HP during the first three stages. At the Fruit ripening stage (BBCH 89), phosphatase activity in the C3 group was 73.48% higher than that in the RP group (Figure 4Ac). Urease activity in the C3 group was significantly higher than that of RP at all stages except flowering, with increases ranging from 1.25- to 1.92-fold compared to RP (Figure 4Ad). Polyphenol oxidase activity in the C3 group was not significantly different from HP but was significantly higher than RP across all stages. During the Fruit ripening stage (BBCH 89), polyphenol oxidase activity in both C3 and HP was 1.95–1.97 times higher than in RP (Figure 4Ae). These results indicate that the application of C3 significantly enhances soil enzyme activities related to nutrient cycling and plant growth, reaching levels comparable to those of healthy plants.

**Figure 5:**
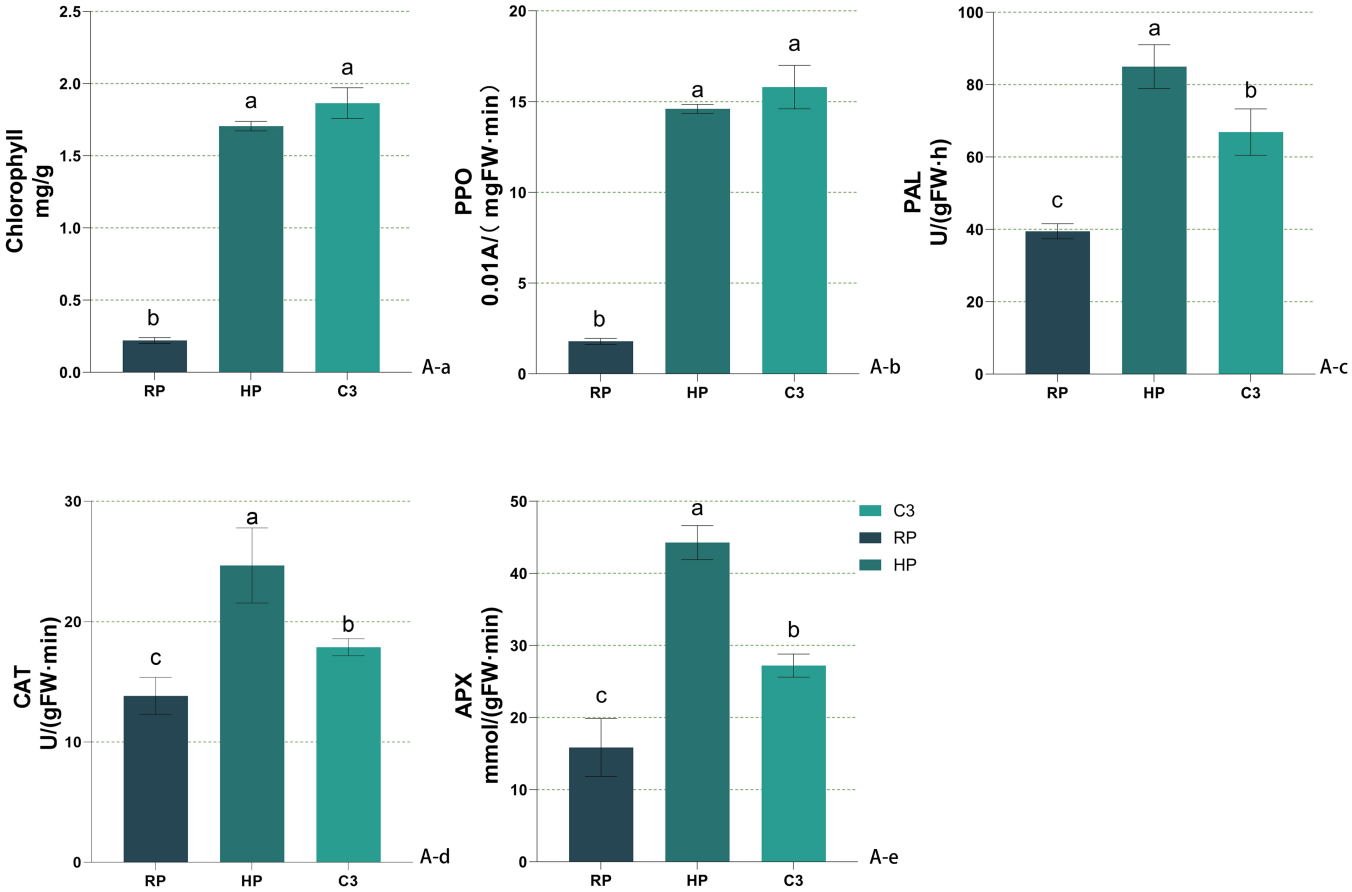
Effects of different treatments on leaves of *Actinidia chinensis*. **(Aa)** Chlorophyll content. **(Ab)** PPO activity. **(Ac)** PAL activity. **(Ad)** CAT activity. **(Ae)** APX activity. In the figure, labels like “A-a” and “B-b” denote specific measured indicators, where capital letters **(A, B)** indicate indicator categories (soil enzymes and soil nutrients), and lowercase letters (a, b, c…) distinguish individual indicators within each category.

Figure 4B presented the physicochemical properties of kiwifruit inter-root soils across different groups. All six soil physicochemical indices were higher in the healthy fruit tree group (HP) compared to the diseased tree group (RP) throughout the kiwifruit growth cycle. The total nitrogen content in the C3 group was 10.73–39.76% higher than that in the RP group across all growth stages and showed no significant difference from the HP group (Figure 4Ba). The nitrate nitrogen content of the C3 group was comparable to HP during germination and flowering but significantly lower than HP during fruit expansion and ripening, while remaining higher than RP throughout the cycle (Figure 4Bb). The ammonium nitrogen content of the C3 group was like HP in the first two stages but exceeded HP in the last two stages (Figure 4Bc). The available phosphorus content in the C3 group was consistently higher than RP across all stages and higher than HP in germination and fruit expansion, while lower than HP during fruit ripening (Figure 4Bd). The available potassium content in the C3 group was significantly higher than RP across all stages, with no significant difference from HP during germination and fruit expansion (Figure 4Be). The organic matter content of the C3 group was significantly higher than RP throughout the cycle, with no significant difference from HP after flowering (Figure 4Bf). The soil pH in the C3 group decreased significantly compared to RP, with reductions of 0.266, 0.113, 0.147, and 0.056 during the four stages, respectively. During germination and fruit expansion, the pH of the C3 group was not significantly different from HP (Figure 4Bg). In Figure 5A, it can be seen that the chlorophyll content (Figure 5Aa) and the activities of resistance enzymes (PPO Figure 5Ab, PAL Figure 5Ac, CAT Figure 5Ad, APX Figure 5Ae) in kiwifruit leaves were significantly higher in the C3 and HP groups compared to the RP group, with no significant differences between the C3 and HP groups, except for catalase activity, which was higher in the HP group. The quality indexes of kiwifruit fruits (Supplementary Table S3) showed that the C3 and HP groups outperformed the RP group overall, except for titratable acid. Specifically, the single fruit weight, soluble sugar, and soluble solids in the C3 group were 29.63, 44.50, and 58.87% higher than those in the RP group, respectively, while soluble protein and vitamin C were 1.88 and 2.84 times higher. The titratable acid content in the C3 group was 0.065% lower, and no significant differences were observed in the fruit type index among the three groups.

## 4 Discussion

This study identified *F. solani* as the primary pathogen responsible for root rot in Xu Xiang kiwifruit through isolation, pathogenicity testing, and molecular identification, consistent with the findings of Song et al. (2023), who reported *F. solani* and *F. breve* as key pathogens in China. Our results validate their observations under different regional conditions and highlight the widespread virulence of *F. solani*, underscoring the need for effective biocontrol strategies such as the application of *B. subtilis* C3. *F. solani* causes severe root damage, disrupts nutrient absorption, leads to leaf chlorosis, reduced fruit quality, and yield loss, and in severe cases, plant death, resulting in significant economic losses for farmers. This finding is consistent with S. Gibert’s initial report of *F. solani* causing root rot in peas in France (Gibert et al., 2022). Pathogenicity tests confirmed that the *F. solani* strain isolated from diseased kiwifruit roots exhibited high virulence, causing leaf wilting and discoloration of root and stem tissues, which are typical symptoms of root rot and consistent with the disease’s characteristics. Molecular sequencing further revealed 99% homology between this strain and *F. solani* (Sequence ID: MG561938) in the GenBank database. These findings align with previous studies, such as those by Xie et al., who identified *F. solani-melongenae* as the pathogen causing root and stem rot in sweet potato (Xie et al., 2022) Pathogenicity tests confirmed that C3 exhibited significant antagonistic activity against the pathogen causing kiwifruit root rot, demonstrating its potential as a biocontrol agent. This was evidenced by the formation of inhibition zones, likely resulting from nutrient competition, ecological niche exclusion, and suppression of spore germination and mycelial growth (Xia et al., 2023). Further analysis revealed that C3 produced various hydrolytic enzymes, including amylase, cellulase, and chitinase, which played a key role in inhibiting pathogen growth, degrading fungal cell walls, and improving soil fertility. These findings are consistent with the study by Ng et al. (2022), which demonstrated that *B. subtilis* enhances soil fertility by increasing soil enzyme activity. The effect of *B. subtilis* C3 on the growth of kiwifruit root rot at seedling stage was studied through pot experiment of C3 to control root rot. The results showed that C3 treatment significantly improved the growth characteristics of the seedlings. Compared to the root rot soil group (RP), the C3 group exhibited significantly higher fresh weight, dry weight, root length, and root-to-shoot ratio, approaching the levels of the healthy soil group (HP). This indicates that C3 improved the rhizosphere soil environment, effectively promoting healthy root development and enhancing the overall growth of kiwifruit plants. Compared to the diseased soil, C3 treatment significantly enhanced the growth of the aboveground parts of the seedlings, with a reduced root-to-shoot ratio, suggesting a shift in nutrient allocation toward the aboveground parts. The study by Ma et al. (2023) emphasized the importance of optimizing fertilization to regulate the root-to-shoot ratio and improve plant growth. Our findings further confirm that *B. subtilis* C3, as a biocontrol fertilizer, could improve disease-affected soils, promote root development, and accelerate aboveground growth, achieving growth conditions comparable to those in healthy soils (Jabborova et al., 2021). Furthermore, C3 enhanced plant resistance and adaptability by improving soil structure, increasing nutrient availability, suppressing pathogen growth, and inducing systemic resistance (El-Saadony et al., 2022). The findings confirm that *B. subtilis* C3 effectively suppresses *F. solani* and enhances soil health, providing a sustainable strategy for managing kiwifruit root rot and improving crop productivity.

*B. subtilis* C3 demonstrates strong potential in managing kiwifruit root rot by targeting *F. solani* through antagonistic activity, enzyme production, and improvement of soil and plant health. In field trials, *B. subtilis* C3 significantly reduced root rot incidence, promoted healthy plant growth, and improved fruit yield and quality. While healthy soil (HP) outperformed other treatments, *B. subtilis* C3 in diseased soil showed results similar to HP, confirming its potential to improve the rhizosphere and alleviate root rot (Han et al., 2021). Application of *B. subtilis* C3 biocontrol fertilizer significantly boosted soil microbial activity in root rot-affected kiwifruit soils (Chen et al., 2023), reaching levels similar to healthy soils (HP) and far surpassing diseased soils (RP). This increase is likely due to the direct introduction of beneficial microorganisms and the fertilizer’s high organic matter content, which provided a carbon source that supported microbial growth and activity (Bueno et al., 2022). In field trials, analysis of the dynamic changes in rhizosphere microbial communities revealed that C3 treatment significantly increased microbial diversity and functional stability. During fruit enlargement and maturation, microbial diversity in the C3-treated soil was significantly higher than in RP soil, approaching HP levels. C3 treatment enhanced the abundance of beneficial microbes (e.g., Bacillus and actinobacteria) while suppressing pathogen expansion. Li et al. (2024). demonstrated that cucumber growth under low calcium stress was improved by *Bacillus sphaericus* QST713, which alleviated stress by enhancing the rhizosphere environment and promoting microbial diversity. C3’s long-term regulation of the soil microbiome provides sustained protection against plant diseases. The microbial diversity index in RP soil was lower than in other treatments, indicating reduced microbial diversity and abundance, with a more uniform structure, which may negatively affect soil fertility conversion. During the germination and fruit enlargement phases, C3 treatment significantly increased the Shannon index, outperforming RP and approaching HP levels (*p* < 0.05), suggesting that C3 extended the fertilization effect and promoted microbial community balance (Pertile et al., 2021). Changes in the Simpson index showed that C3 facilitated the recovery of microbial species richness to normal levels. Although microbial diversity in C3-treated soil was slightly lower than in HP during the fruit enlargement phase, it was consistent with the AWCD activity results, which showed that microbial activity in C3 was slightly lower than HP. The McIntosh index significantly increased during the germination phase, indicating that C3 improved microbial community uniformity (Chen et al., 2021). During the transition from fruit enlargement to maturity, both HP and C3 treatments showed a decline in uniformity, possibly due to high temperatures affecting microbial community stability. High-throughput sequencing results revealed that C3 significantly increased microbial diversity and abundance, approaching HP levels, while RP showed pathogen dominance. The study suggests that higher microbial diversity enhances soil disease resistance, and C3 promotes a healthier soil environment for kiwifruit plants by improving the microbial community and supporting the growth of functional microbes (Ahsan et al., 2024). *B. subtilis* C3 demonstrates strong potential in managing kiwifruit root rot by targeting *F. solani* through antagonistic activity, enzyme production, and improvement of soil and plant health.

*B. subtilis* C3 significantly improved soil enzyme activity and physicochemical properties, as evidenced by pot and field experiments. The findings underscore its critical role in restoring soil fertility and promoting plant growth in root rot-affected soils. Microbial formulations can produce various enzymes that facilitate the degradation of organic matter in the soil. Soil enzyme activity is a crucial indicator for assessing nutrient cycling and soil fertility (Ali et al., 2024). The application of C3 significantly enhanced the activity of key soil enzymes, including sucrase, phosphatase, and protease, which play vital roles in the transformation and release of carbon, phosphorus, and nitrogen (Bai et al., 2022). These enzymes are important indicators for assessing nutrient cycling and soil fertility (Tuo et al., 2023). Particularly during the fruit enlargement phase, enzyme activity in C3-treated soil was comparable to that of healthy soil (HP) and significantly higher than in diseased soil (RP). This suggests that C3 improves soil enzyme activity, enhances nutrient availability, and promotes plant growth (Padró et al., 2021). Additionally, enzymes such as phosphatase, polyphenol oxidase, and sucrase are closely associated with the occurrence of root rot, indicating that C3 may contribute to improving soil health and enhancing disease resistance (Rashad et al., 2022). Nutrients in the soil primarily exist in insoluble forms, making them unavailable for direct absorption by plants (Das et al., 2022). Studies have shown that microbial formulations can convert these nutrients into soluble forms, enhancing their availability to plants and improving soil fertility (Olaniyan et al., 2022). C3 treatment also significantly improved the physico-chemical properties of soil affected by root rot (Deng et al., 2025). Compared to the RP group, the C3 treatment significantly increased soil organic matter, carbon-to-nitrogen ratio, and available nutrients, with soil pH also trending toward neutrality (Yu et al., 2024). These changes contribute to the restoration of soil health, providing a better growing environment for plant roots (Ding et al., 2024). This is consistent with previous studies showing that *B. subtilis* promotes plant health by improving soil structure and physico-chemical properties (Ku et al., 2021). The application of *B. subtilis* C3 significantly enhances soil enzyme activities and physico-chemical properties, improving nutrient availability and fostering a healthier plant growth environment. These results highlight C3’s potential as an effective microbial amendment for managing root rot and enhancing soil health, reinforcing its role in sustainable agriculture.

PAL, PPO, CAT, and APX are essential for kiwifruit defense against biotic and abiotic stresses, with higher enzyme activities correlating with stronger disease resistance. In contrast, root rot-infected plants show reduced enzyme activities and increased susceptibility. *B. subtilis* C3 induces these defense enzymes, enhancing systemic resistance and disease tolerance. Phenylalanine ammonia-lyase (PAL), polyphenol oxidase (PPO), catalase (CAT) (Zhang J. et al., 2022), and ascorbate peroxidase (APX) play key roles in plant defense against pathogens and environmental stress (Rajput et al., 2021). In this study, healthy kiwifruit exhibited higher chlorophyll content and enzyme activity, while plants affected by root rot showed reduced enzyme activity, making them more susceptible to pathogen invasion (Mehmood et al., 2021). *B. subtilis* C3 can induce the production of plant defense enzymes and enhance systemic resistance, which is consistent with our findings (Ayaz et al., 2024). C3 treatment significantly increased chlorophyll content and defense enzyme activity in diseased plant leaves, enhancing their disease resistance (Lastochkina, 2019). Additionally, C3 treatment significantly improved fruit quality by increasing fruit hardness, soluble sugars, vitamin C, and total soluble solids, while reducing titratable acidity (Fan et al., 2023). Compared to the RP group, the fruit quality in the C3 treatment group was close to that of the HP group, indicating that C3 not only controls root rot but also indirectly enhances fruit commercial value by improving soil and plant health (Li B. et al., 2023). C3 treatment not only mitigated root rot but also improved fruit quality by enhancing attributes such as hardness, soluble sugars, vitamin C, and total soluble solids, while reducing acidity. The fruit quality of C3-treated plants rivaled healthy controls, highlighting its potential to improve soil and plant health and enhance the fruit’s commercial value.

## 5 Conclusion

In summary, this study successfully identified *Fusarium solani* as the pathogenic fungus responsible for root rot in Xuxiang kiwifruit. Moreover, C3 showed significant antagonistic activity against pathogenic bacteria and demonstrated strong biological control potential. C3 effectively controls root rot by antagonizing the pathogen, secreting antimicrobial enzymes, promoting seedling growth, and inducing the activity of defense enzymes such as phenylalanine deaminase and polyphenol oxidase. Pot experiments and field experiments confirmed that the application of C3 improved rhizosphere soil quality, increased microbial diversity, enhanced soil enzyme activity, and restored soil fertility. In addition, C3 treatment increased the activity of resistant enzymes in kiwifruit leaves and improved fruit quality, indicating its potential as a biological control agent in root rot control and improving kiwifruit health and productivity.

## Data Availability

The datasets presented in this article are not readily available because the datasets generated and analyzed during the current study are available from the corresponding author upon reasonable request. Usage restrictions apply to protect ongoing research and participant privacy; thus, datasets cannot be publicly shared but are accessible under specific conditions agreed upon by the authors. Requests to access the datasets should be directed to songxiaolong0413@gmail.com.
