# Supplementary material for: Ecological effects of B. subtilis C3 in kiwifruit rhizosphere soil and its prevention and control against root rot disease
Source: Front Microbiol. 2025 Aug 21;16:1623463. doi: 10.3389/fmicb.2025.1623463 (PMC12408652; doi:10.3389/fmicb.2025.1623463)
Supplement: Supplementary file 1 [file Table_1.docx]

Supplementary Material

# Supplementary Tables and Figures

**Table S1 Enzyme Production of *Bacillus subtilis***

| Enzyme production | Enzyme activity (U/mL) |
| --- | --- |
| Amylase | 2.080±0.021 |
| Cellulase | 180.136±9.646 |
| Protease | 110.905±2.767 |
| Chitinase | 3.253±0.199 |
| phytases | 12.486±0.379 |
| Phosphatase | 1.812±0.047 |

**Table S2 The ability of C3 to secrete hormones**

| Hormone | Content |
| --- | --- |
| IAA (mg/L) | 24.263±0.864 |
| GA (μg /ml) | 0.041±0.013 |

| Treatment | Fruit Weight kg | Soluble Protein mg/g | Chlorophyll mg/100g | Soluble Sugar mg/g | Fruit Firmness kg/cm^2^ | Vitamin C mg/100g | Soluble  Solids  % | Titratable acid  % | Fruit Features |
| --- | --- | --- | --- | --- | --- | --- | --- | --- | --- |
| RP | 0.081±0.011c | 6.501±0.302b | 0.254±0.013b | 43.460±2.802c | 11.373±0.720b | 62.707±1.536b | 9.143±0.051b | 0.768±0.063a | 1.013±0.010a |
| HP | 0.125±0.015a | 13.155±0.567a | 0.442±0.030a | 71.630±1.553a | 14.843±0.604a | 162.285±7.102a | 13.337±0.076a | 0.623±0.046b | 1.088±0.063a |
| C3 | 0.105±0.016b | 12.231±0.294a | 0.266±0.019b | 62.800±5.191b | 13.376±0.789a | 178.100±10.483a | 14.527±0.344a | 0.703±0.030a | 1.107±0.075a |

**Table S4 Effects of different treatments on the fruit quality of kiwifruit**

# Supplementary Figures


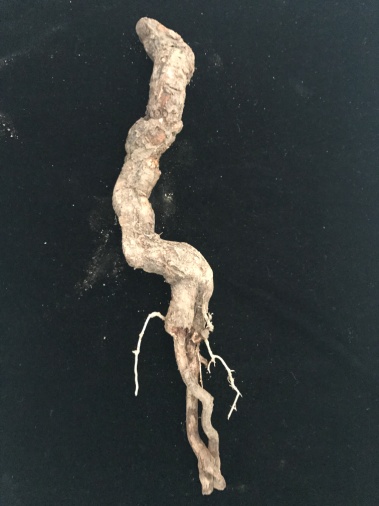


**Fig.S1 Root cause of kiwifruit root rot.**


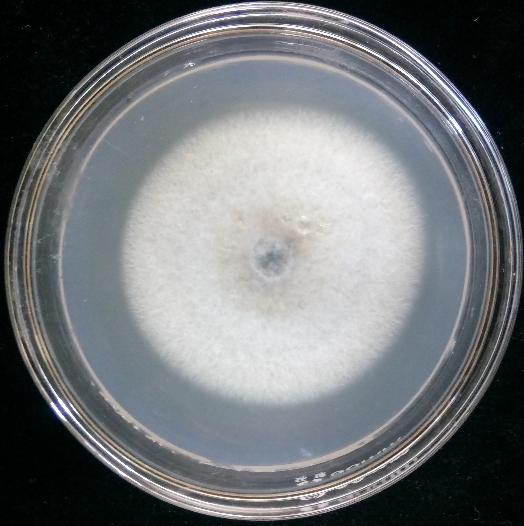


**Fig.S2 *Fusarium solani* isolated from kiwifruit root rot on PDA plate**


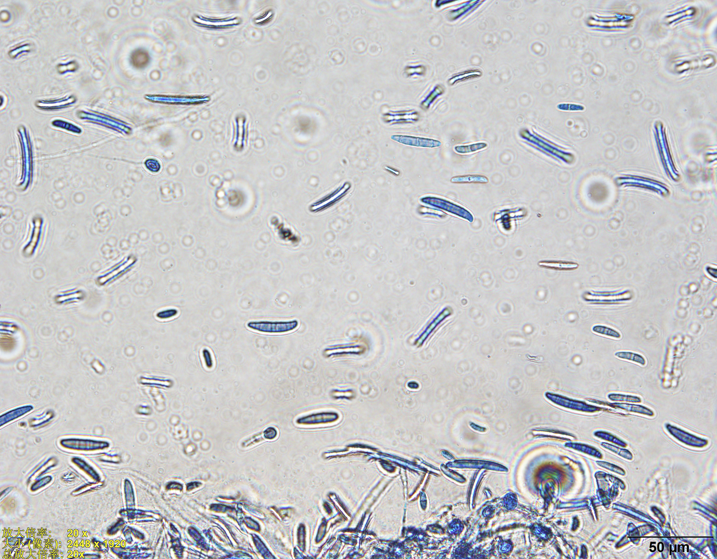


**Fig.S3 *Fusarium solani* observed under a light microscope (scale bar = 50 μm)**


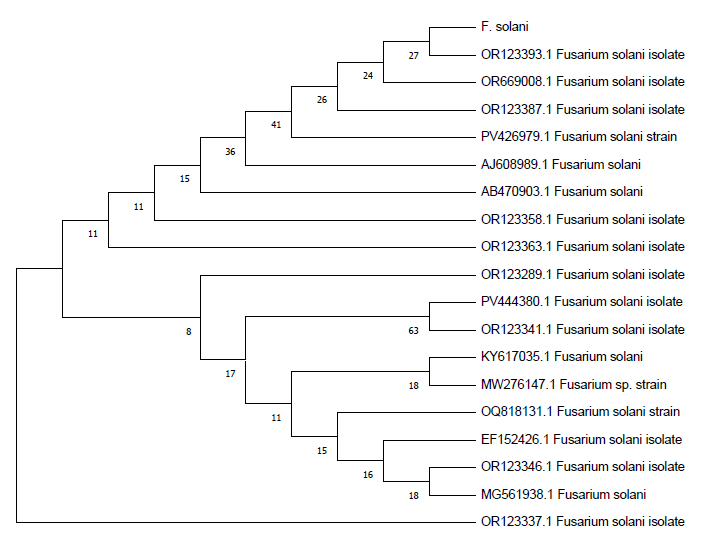


**Fig.S4 Phylogenetic tree of *Fusarium solani* isolates associated with kiwifruit root rot based on ITS region sequences**


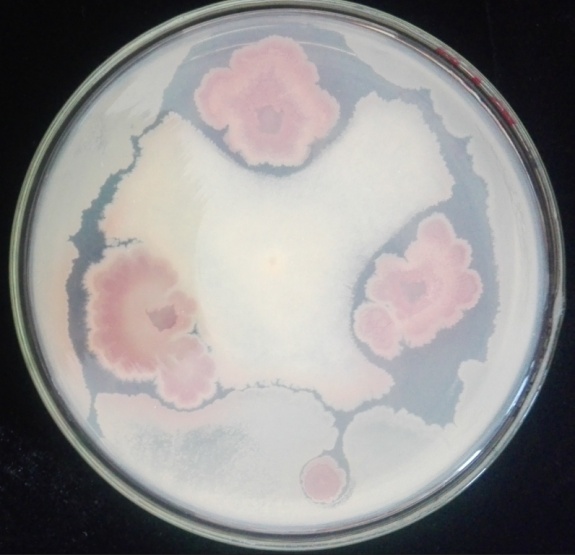


**Fig.S5 Root causes kiwifruit root rot.**

**Fig.S6 Phylogenetic tree based on rDNA sequence of strain 16S**

**
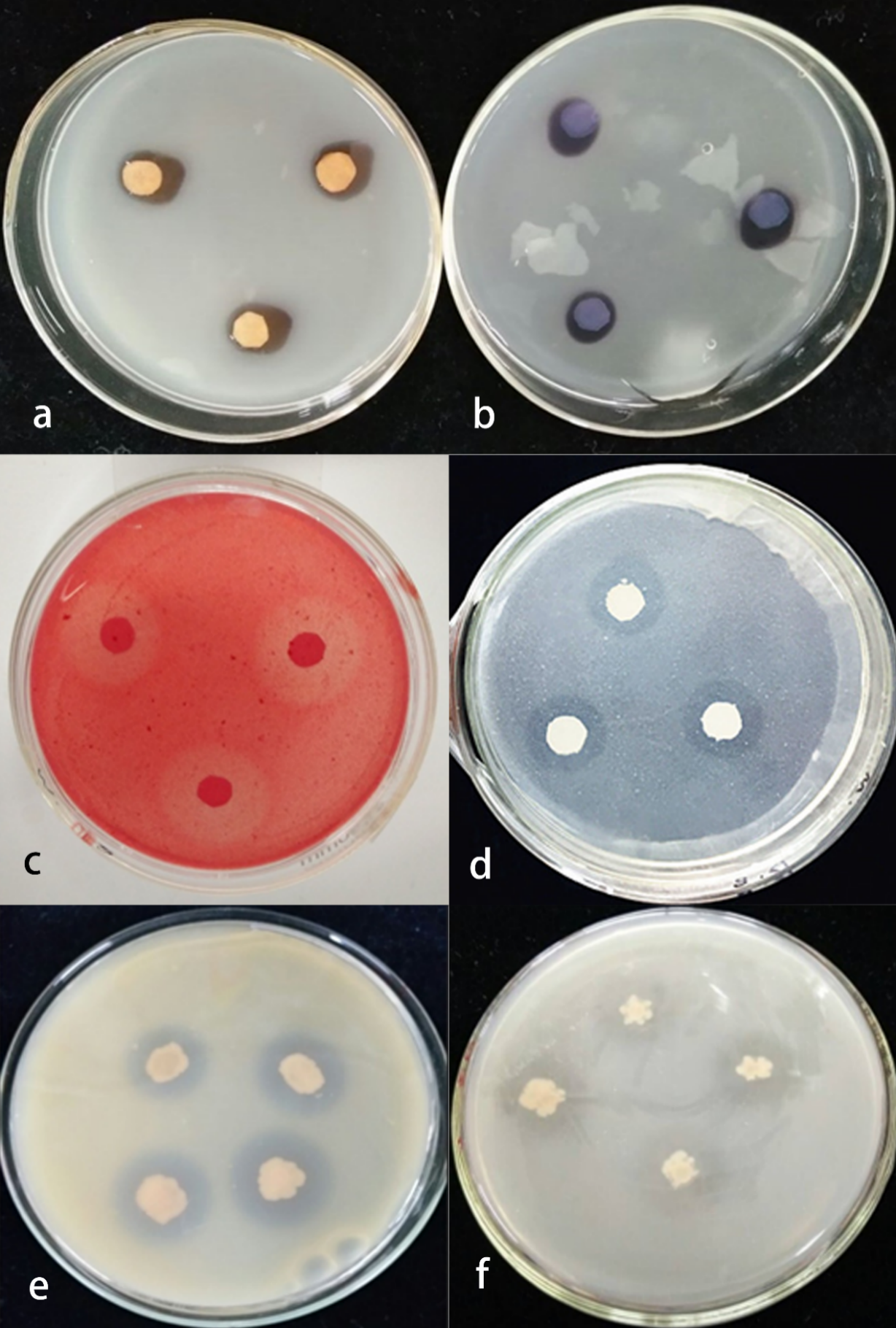
Fig.S7 *Bacillus subtilis* C3 producing enzyme qualitative plate** **Figure S7.**

*Bacillus subtilis* C3 enzyme production on starch agar. Fig.S7-a shows starch agar plates with C3 fermentation supernatant dropped on the left and sterile water on the right. After iodine staining, the plate in Fig.S7-b did not turn blue, indicating that strain C3 has amylase-producing activity. Fig.S7c–d Qualitative assay plates showing cellulose- and protease-producing activity of *Bacillus subtilis* C3. Fig.S7e–f. Qualitative assay plates showing phosphatase and chitinase activity produced by *Bacillus subtilis* C3.


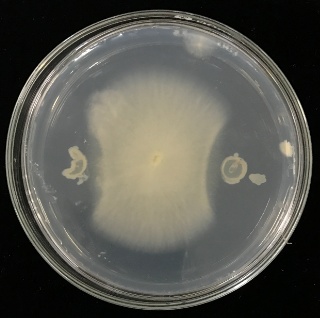


**Fig.S8 Antagonistic effect on pathogenic bacteria strain *Bacillus subtilis* C3**


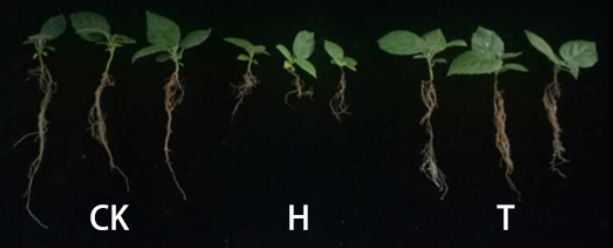


**Fig.S9 Effects of different treatments on the seedlings of kiwifruit seedlings**
